# Efficacy and Safety of Immunosuppressive Therapy for PBC–AIH Overlap Syndrome Accompanied by Decompensated Cirrhosis: A Real-World Study

**DOI:** 10.1155/2018/1965492

**Published:** 2018-08-02

**Authors:** Xiaoli Fan, Yongjun Zhu, Ruoting Men, Maoyao Wen, Yi Shen, Changli Lu, Li Yang

**Affiliations:** ^1^Department of Gastroenterology & Hepatology, West China Hospital, Sichuan University, Chengdu, Sichuan 610041, China; ^2^Department of Pathology, West China Hospital, Sichuan University, Chengdu, Sichuan 610041, China

## Abstract

**Aim:**

To explore the efficacy and safety of immunosuppressive therapy for the treatment of primary biliary cirrhosis-autoimmune hepatitis (PBC-AIH) overlap syndrome accompanied by decompensated cirrhosis.

**Methods:**

A cohort study was performed to evaluate the usefulness of immunosuppressive therapy in this unique group. This cohort study was performed between October 2013 and June 2017 and included 28 biopsy-proven patients diagnosed according to the Paris criteria. The therapies included ursodeoxycholic acid (UDCA) alone (N=14) or in combination with immunosuppression (IS) therapy (N=14). The primary endpoints were biochemical remission, liver-related adverse events, transplant-free survival, and drug side-effects.

**Results:**

The frequency of biochemical remission for the AIH features was significantly higher in the UDCA+IS group than in the UDCA-only group (60.0 versus 9.1%, P=0.024) after 12 months of therapy but not after 3 and 6 months (28.6 versus 0%, P=0.165; 35.7 versus 7.1%, P=0.098). The rates of liver-related adverse events were lower in the combined group (2/14 versus 9/14, P=0.018). The Kaplan-Meier estimate showed that the transplant-free survival was distinct between the two groups (P=0.019). In the UDCA+IS group, mild and transient leukopenia occurred in two patients receiving azathioprine (AZA), and an infection was observed in one patient receiving mycophenolate mofetil (MMF).

**Conclusions:**

PBC-AIH patients with decompensated cirrhosis receiving a combination of UDCA and immunosuppressors presented with higher biochemical remission rates and experienced fewer liver-related adverse events, implying that the combined treatment might be a better therapeutic option for strictly defined decompensated PBC-AIH overlap syndrome.

## 1. Introduction

Autoimmune liver disease (AILD) comprises a group of immune-mediated liver diseases that include autoimmune hepatitis (AIH), primary biliary cirrhosis (PBC), and primary sclerosing cholangitis (PSC) [1]. The occurrence of overlapping syndromes at different disease stages (so-called overlap syndromes) is not rare, with PBC-AIH overlap syndrome being the most common [2]. The prevalence of PBC-AIH overlap syndrome is approximately 8–10% in adult patients with either PBC or AIH [3, 4], and this low incidence contributes to imprecise diagnostic criteria; furthermore, no standard therapy is currently available. According to the most recent guidelines based on the results of small studies, a combination of ursodeoxycholic acid (UDCA) and immunosuppressants is often recommended for PBC-AIH in clinical practice [5–7].

Risk stratification was recently performed by a panel of experts on the topic of cirrhosis [8]. The survival rates of compensated and decompensated cirrhosis are strikingly different, and the median survival time of the latter may be less than 2 years [9, 10]. PBC-AIH patients presenting with already advanced cirrhosis may die in the early phase of treatment because of complications related to immunosuppression; however, there are no data supporting this finding [11]. Hence, the present study was undertaken to analyse a single-centre cohort of PBC-AIH patients with decompensated cirrhosis. The present study was initiated to determine whether immunosuppressive therapy could be used in a cohort of decompensated PBC-AIH patients to obtain a biochemical response and to control the disease progression and the cost in terms of adverse reactions.

## 2. Materials and Methods

### 2.1. Inclusion Criteria

West China Hospital is a 4300-bed tertiary teaching hospital affiliated with Sichuan University. The hospital has a liver transplant unit (Liver Transplantation Centre, West China Hospital) and is the leading hospital in the western areas of China.

Cases were prospectively recruited between October 2013 and June 2017 using an electronic database of AILD, which was established in October 2013. Twenty-eight consecutive patients with PBC-AIH with decompensated cirrhosis strictly according to the Paris criteria were incorporated. The study was approved by the Ethics Committee of West China Hospital.

As recommended by the Paris criteria, the patients were diagnosed with PBC when they met 2 or more of the following diagnostic criteria: (1) the presence of anti-mitochondrial antibodies (AMA), (2) an alkaline phosphatase (ALP) level at least 2-fold the upper normal limit (UNL) or a gamma-glutamyl transpeptidase (GGT) level at least 5-fold the UNL, and (3) a liver biopsy specimen exhibiting florid bile duct lesions. Patients were diagnosed with AIH when they met 2 or more of the following criteria: (1) an alanine aminotransferase (ALT) level at least 5-fold the UNL, (2) serum IgG at least 2-fold the UNL or a positive test for smooth muscle antibodies (SMA), and (3) a liver biopsy exhibiting moderate or severe periportal or periseptal lymphocytic piecemeal necrosis. In our setting, only simultaneous forms of PBC-AIH overlap syndrome were enrolled, because fewer patients had the consecutive forms, which might have raised different diagnostic and therapeutic issues [12, 13]. Patients with viral hepatitis, nonalcoholic steatohepatitis, drug-induced liver disease, Wilson's disease, or other causes of liver damage were excluded through a careful history analysis and evaluation. Overlap with suspected PSC and acute severe AIH, as defined according to the proposed criteria, was also excluded [14].

Cirrhosis was diagnosed according to the histological analysis, unequivocal imaging, or endoscopic examination [15]. Decompensation was diagnosed by the presence of clinical complications, including ascites, variceal hemorrhage, and hepatic encephalopathy (HE) [8].

### 2.2. Clinical and Laboratory Analyses

The biochemical, serological, radiological, and histological data, treatment strategies, and outcomes were recorded for the patients with PBC-AIH with decompensated cirrhosis. The laboratory measurements included total bilirubin (TBIL), ALT, aspartate aminotransferase (AST), ALP, GGT, albumin (ALB), globulin (GLB), IgG, IgM, antinuclear antibody (ANA), liver-kidney microsomal antibody (LKM), soluble liver antigen (SLA), antibody against liver cytosol type 1 antigen (LC-1), routine blood measurements, and noninvasive hepatic fibrosis parameters. Child-Pugh scores were collected at baseline. All parameters were examined in the Department of Laboratory Medicine of West China Hospital, which was certified by the College of American Pathologists (CAP).

The imaging tests included ultrasonography (US), computed tomography (CT), and/or magnetic resonance imaging (MRI).

All patients underwent follow-up, including clinical and laboratory evaluations, every 1–3 months.

### 2.3. Treatment

A total of 28 consecutive decompensated PBC-AIH patients were enrolled in this open, real-world, observational study. Because the optimal type of treatment was not known, the managing physician was free to decide whether he would treat the patients with UDCA alone or combined with immunosuppressants. The patients treated with UDCA alone received a 13–15 mg/kg/d dose. In the UDCA+IS group (N=14), the patients were given an initial dose of 12-40 mg/d of methylprednisolone, simultaneously. Induction therapy was response-guided and individualized for the 14 patients and was gradually reduced. Azathioprine (AZA) (50-100 mg/d) was combined with UDCA and steroids for 12 patients in the UDCA+IS group, whereas mycophenolate mofetil (MMF) was combined with UDCA and steroids for one patient when the total bilirubin level was above 100 *μ*mol/L and the other one was merely given UDCA and steroids. Proton pump inhibitors (PPIs) were used to prevent peptic ulcers or bleeding during corticosteroid reduction, and vitamin D and calcium were introduced to prevent osteoporosis and fractures.

Prior to the initiation of corticosteroid therapy, an absence of infection was confirmed by negative cultures of blood samples, ascites fluids, and urine specimens and chest X-ray or CT. Common complications were prevented and treated according to accepted clinical management guidelines.

### 2.4. Pathological Examination

Liver samples were acquired by ultrasound-guided percutaneous needle biopsy of the liver. The tissues were fixed in 10% formaldehyde (Kelong, China), embedded in paraffin, and used for haematoxylin and eosin (H&E) staining, Masson trichrome staining, special staining, and immunohistochemical staining to examine the histological characteristics. Finally, two pathologists (Changli Lu and Jianping Liu) from the Pathology Department of West China Hospital (certified by CAP) interpreted the samples. Diagnostic pathological changes in AIH were recorded, including interface hepatitis, lymphoplasmacytic infiltrate, hepatocyte resetting, and emperipolesis. Pathological changes used for the diagnosis of PBC included florid duct lesion, bile duct damage, ductular proliferation, and cholestasis. The activity grade (G0–4) and fibrosis stage (S0–4) were assessed, according to the Scheuer system [16–19].

### 2.5. Outcomes Assessment

The primary effectiveness assessment was biochemical remission of AIH features (normalization of transaminases and IgG after starting therapy, as determined using existing guidelines). Liver-related adverse events were determined if the patients experienced the following: (1) worsening of existing hepatic decompensation, (2) a new decompensation event other than the event present at the initiation of therapy, (3) liver failure, or (4) transplantation or death attributable to decompensation events. The transplant-free survival was compared additionally. The evaluation of drug side-effects included diarrhoea, steroid-specific side effects (i.e., osteoporosis, fractures, moon face, or acne), infection, and myelosuppression. All safety assessments were performed for the total patient cohort. Patients were followed until May 2018.

### 2.6. Statistical Analysis

Continuous data are presented as medians and quartiles, and categorical data are expressed as percentages. The Mann–Whitney U test was used for comparisons of continuous variables. The Chi-square test or Fisher's exact test was used to analyse differences in categorical variables between two independent groups where appropriate. A P-value <0.05 was considered significant. Data processing was performed with the SPSS software package (SPSS version 24.0 for Windows, IBM Corp., Armonk, NY, USA).

## 3. Results

### 3.1. Basic Information

**Figure 1:**
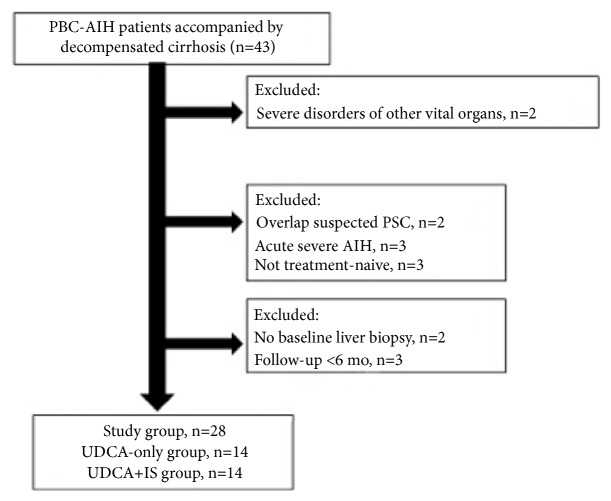
Study flowchart for patient inclusion.

A total of 28 patients diagnosed with PBC-AIH accompanied by decompensated cirrhosis were enrolled in the cohort study (Figure 1).  Table 1 presents the clinical and biochemical characteristics of the two groups. For the UDCA alone group, the median age at treatment initiation was 60.0 (51.3, 61.3) years, and the female-to-male ratio was 13:1. For the UDCA+IS group, the median age was 48.0 (42.5, 53.5) years, and the female-to-male ratio was 12:2. AMA was positive in 64.2% of patients (9 of 14) in the both the UDCA alone group and UDCA+IS group (P>0.999). SLA, LC-1, and LKM were negative in the enrolled patients. At the time of study inclusion, the patients who did not receive immunosuppressants had lower total bilirubin (TBIL) levels (29.0 versus 38.9 IU/L, P=0.035) and higher albumin (ALB) levels (37.6 versus 32.3 IU/L, P=0.031) than the combined group. All patients were diagnosed with decompensated cirrhosis prior to treatment (Table 1).

The common clinical symptoms are shown in Table 2. Jaundice and ventosity were the two most common symptoms in all patients. The symptoms at presentation were similar in the two groups.

### 3.2. Histological Features in the Study Cohort

**Figure 2:**
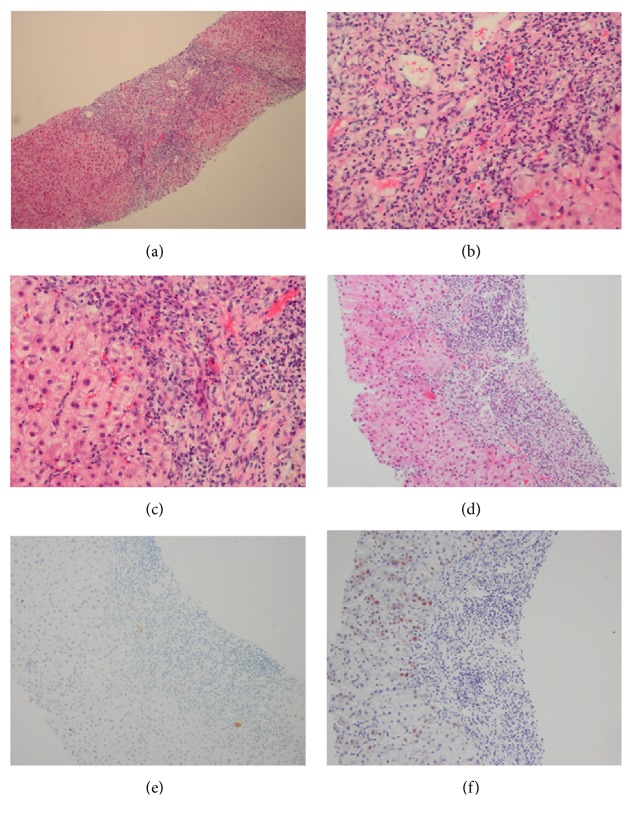
Histological features of decompensated PBC-AIH patients. (a) Bridging necrosis and moderate to severe interface hepatitis (×100, HE staining); (b) prominent interface hepatitis with numerous plasma cells (×400, HE staining); (c) typical rosetting of hepatocytes in the area of interface hepatitis (×400, HE staining); (d), (e), (f) interlobular bile duct loss without a significant ductular reaction (×200, HE staining, CK7 staining, and copper staining in sequence).

All 28 patients had biopsy specimens available at diagnosis and presented with a typical picture of AIH features, with moderate to severe interface hepatitis and lymphocytic infiltrates, as well as PBC features. Severe interface hepatitis was only observed in 2 patients in the combined group. Hepatocellular rosette formation was noted in 8 and 10 patients in the two groups, respectively. Lymphoplasmacytic infiltrate was found in most patients, whereas emperipolesis was not observed. Bile duct damage was found in 13 and 10 patients in the two groups, whereas ductopenia was observed in 9 and 6 patients, respectively. Ductular proliferation, which was revealed by staining for cytokeratin 7, was observed in 11 and 9 patients in the two groups, respectively (Table 3).  Figure 2 presents the features of a biopsy specimen from a patient who received UDCA plus immunosuppressant treatment.

### 3.3. Treatment Response

Table 4 shows the treatment response rates for both patient groups. At the end of the study, 42.9% (12/28) of the patients reached complete biochemical remission. Overall, the response rates after 3, 6, and 12 months of therapy were 14.2% (4/28), 21.4% (6/28), and 33.3% (7/28), respectively. The remission rate after 12 months was significantly higher in the combined group than in the UDCA-only group (60.0 versus 9.1%, P=0.024). Although not statistically significant, the biochemical remission rates seem to be higher in the combined group after 3 and 6 months of therapy (28.6 versus 0%, P=0.098 and 35.7 versus 7.1%, P=0.165).

The symptoms of 12/14 (85.7%) of patients in the combined group have improved significantly, while the rate of symptom improvement was 71.4% (P=0.353).

### 3.4. Liver-Related Adverse Events and Transplant Free Survival Period

Table 5 shows the clinical outcomes at the end of the study. During the study period, the rates of liver-related adverse events were 64.3% (9 of 14) and 14.3% (2 of 14) in the UDCA alone group and the combination group, respectively (*P*=0.018). Thus, a total of 11 patients experienced liver-related adverse events during the follow-up. The median follow-up time was 18.0 (13.3, 20.8) months and was similar for patients in the UDCA-only group and the combined group [20.0 (13.5, 26.8) months, respectively (P=0.427]. To date, relapses during maintenance therapy have not been observed, and immunosuppression has not been withdrawn.

**Figure 3:**
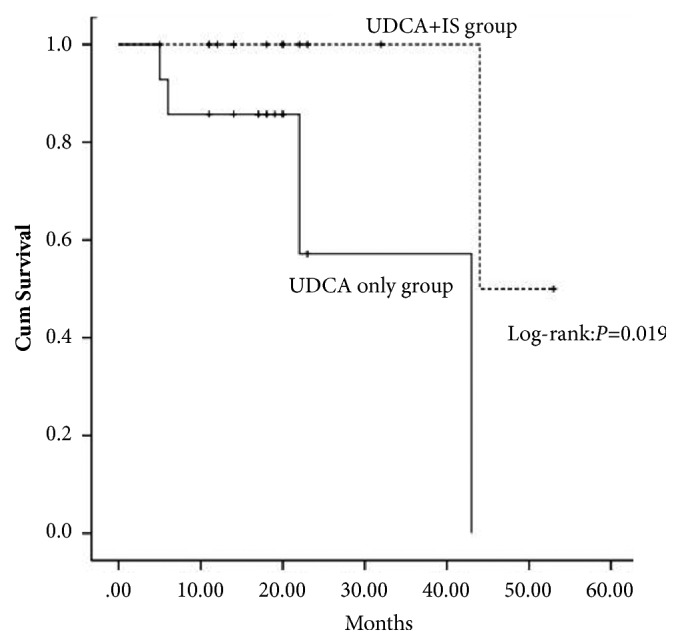
Transplant-free survival between the two groups (log-rank, P = 0.019).

Liver transplantations were more commonly observed in the UDCA-only group and the combined group (4/14 vs 1/14, P=0.326). No patient died till the end of follow-up. The reasons for liver transplantation were cholestasis (N=3), esophagogastric hemorrhage (N=1), and refractory ascites (N=1). In the 5 patients who received liver transplantations in the two groups, none died posttransplantation. The Kaplan-Meier estimate showed that the transplant-free survival was distinct between the two groups (P=0.019) (Figure 3).

### 3.5. Drug Side-Effects

Seven patients experienced transient diarrhoea after UDCA treatment. Corticosteroid-related side effects were noted in 35.7% (5 of 14) of the patients in the combined group. Most of these effects were mild, steroid-specific side effects, such as acne and moon face, and most resolved after dose reduction. Two patients in the UDCA+IS group experienced mild leukopenia (white blood count values of 3.0 × 10^9^/L and 2.49 × 10^9^/L) during the first 60 days of AZA treatment. AZA was not discontinued, because the white blood counts increased gradually and thereafter remained stable. A mild urinary tract infection occurred in one patient who received MMF during the first 60 days of AZA treatment.

## 4. Discussion

This cohort study was the first to assess the real-world effectiveness and safety of immunosuppressors in decompensated PBC-AIH patients. Six patients (60%) receiving UDCA plus immunosuppressors achieved biochemical remission, whereas only one patient (9.1%) achieved biochemical remission 12 months after beginning therapy. Patients treated with a combination of UDCA and immunosuppressors experienced fewer liver-related adverse events and obtained longer transplant-free survival, implying that the natural history and progression of decompensated PBC-AIH may also be haltered and averted.

Biochemical remission is a predictor of histologic outcome in PBC-AIH and may postpone the progression of cirrhosis [20]. In the present study, biochemical remission occurred in 60.0% of patients treated with a combination of UDCA and immunosuppression. Our data were consistent with the results from other studies, although these studies did not investigate advanced cirrhosis. A multicentre retrospective study with 88 patients with PBC-AIH found that the combination of UDCA and immunosuppression was effective in 73% of patients who were not previously treated or did not respond to UDCA alone [20]. Chazouilleres et al. found that fibrosis progression occurred more frequently in noncirrhotic patients under UDCA monotherapy (4/8) than under combined therapy (0/6) (*P*=0.04) [21]. Similar results were obtained in a recent meta-analysis, which found that combination therapy with UDCA and corticosteroids was more effective than UDCA alone [6]. In our study, most of the biochemical and immune parameters improved dramatically in both groups after treatment, whereas the immune variable IgG, which is a hallmark of liver inflammation and the treatment response [5], did not decrease in the UDCA group. However, the TBIL values, which were used as one element to evaluate AIH remission [22], did not significantly differ before and after treatment in either group (data not shown). Early studies have suggested that UDCA alone can help achieve biochemical and histological improvements in PBC-AIH patients, but the patient groups of the studies being compared did not focus on PBC–AIH overlap syndrome accompanied by decompensated cirrhosis [23, 24]. Hence, our data, similar to data from other studies, emphasize that the administration of immunosuppressors aids the hepatic immune response in this group of patients.

The median survival time for decompensated cirrhosis may be less than 2 years [9, 10]. Recent EASL guidelines proposed that treatment was probably no longer indicated in AIH patients with decompensated cirrhosis unless they had a high inflammatory score on the liver biopsy. However, limited studies have offered real-world data for the responses, outcomes, and side effects in AIH or PBC-AIH patients with decompensated cirrhosis [5, 25]. In the present study, the data showed a benefit on the progression of advanced cirrhosis in the UDCA+IS group during the observation period (median, 15.0 months). This result was in line with another study. Wang et al. found that immunosuppression treatment helped 62.5% of AIH patients with decompensated cirrhosis revert to compensated cirrhosis and that the rate of transplant-free survival was significantly greater in patients who received corticosteroids compared to those who did not [25]. Although the relationship between PBC-AIH overlap syndrome and AIH alone is complex, their results implied that immunosuppressive treatment could interrupt the progression of autoimmune-mediated advanced cirrhosis by controlling the hepatic immune response. In our study, all the patients were diagnosed according to Paris criteria; that is, PBC patients were diagnosed with AIH when they met 2 or more of the criteria, which meant that our patients were with histological features of active liver inflammation, rather than quiet or burnt-out cirrhosis. In that situation, more emphasis would be needed on the treat complications to prolong the transplant-free survival. Hence, individualized protocols are needed for different clinical situations. However, further prospective studies with more patients and long-term follow-ups may provide more robust evidence.

Prior to starting therapy, the risks and benefits of immunosuppressive treatment must be weighed. In our study, 12 patients (85.7%) received an initial methylprednisolone dose of 24 mg/d that was rapidly tapered according to the treatment response. The methylprednisolone doses for the other two patients were 12 mg/d and 48 mg/d. No serious side-effects of methylprednisolone were observed in our study, which was mostly attributed to our positive strategies used for tapering, preventive medications, and monitoring. In the 12 patients who received AZA as an adjuvant drug, transient leukopenia occurred in 2 patients (14.2%). Our data were not consistent with Heneghan et al. [26], who found that advanced fibrosis but not the thiopurine methyltransferase (TPMT) genotype or activity predicted azathioprine toxicity in AIH patients. In our patients,* NUDT15* (i.e., rs116855232) and* TPMT* (i.e., rs1142345) SNPs were genotyped using a real-time PCR method. No TPMT genetic variants were genotyped in these 12 patients, but heterozygous* NUDT15 *R139C genotypes were found in the 2 patients who experienced leukopenia (data not shown). Hence, adjusting the azathioprine dosage may be considered according to the rs116855232 genotype rather than advanced fibrosis or cirrhosis. Hence, the rate of leukopenia was relatively low and acceptable. However, whether the* TPMT* and* NUDT *genotypes were associated with leukopenia in these PBC-AIH patients is unknown. Also, the infection rate was 7.1%, which was in line with Wang et al. (10.9%) [25].

The present study has some limitations. First, this investigation was a single-centre cohort study. Given this experimental approach, avoiding confounding factors was relatively difficult, and some data were not available. Second, because decompensated cirrhosis is an uncommon and advanced presentation of this disease, obtaining larger sample sizes is difficult in real-word studies. The current lack of controlled trials makes the results of small studies on rare diseases helpful and informative. Furthermore, because the mortality rate is as high as 85% over 5 years in patients with decompensation who do not receive a liver transplant, conducting a prospective study with extended follow-up can be very difficult. Third, no histological evaluations were performed after therapy to confirm the histological validity of this study.

In conclusion, we found that PBC-AIH patients with decompensated cirrhosis receiving a combination of UDCA and immunosuppressors presented higher biochemical remission rates, fewer liver-related adverse events, and longer transplant-free survival, implying that combined treatment might be a better therapeutic option for strictly defined PBC-AIH overlap syndrome accompanied by decompensated cirrhosis.

## Figures and Tables

**Table 1:** Demographic and clinical features, laboratory parameters, prognostic scores, and decompensation characteristics between the two groups.

**Variables**	**UDCA-only group (N=14)**	**UDCA+IS group (N=14)**	***P* value**
Age at entry (years)	60.0 (51.3, 61.3)	48.0 (42.5, 53.5)	0.024
Gender (F/M)	13/1	12/2	>0.999
TBIL, *μ*mol/L	29.0 (23.2, 41.1)	38.9 (35.2, 127.1)	0.035
ALT, IU/L	66.5 (44.8, 127.0)	112.0 (45.7, 174.5)	0.401
AST, IU/L	103.5 (78.5, 129.8)	170.0 (91.0, 212.5)	0.401
ALP, IU/L	349.5 (257.8, 519.0)	294.0 (196.5, 430.5)	0.285
GGT, IU/L	268.5 (157.0, 656.3)	229.5 (51.8, 293.5)	0.285
ALB, g/L	37.6 (31.8, 39.9)	32.3 (29.9, 35.5)	0.031
GLB, g/L	43.4 (37.4, 47.1)	45.4 (35.3, 50.2)	0.511
INR	1.1 (1.0, 1.2)	1.1 (1.0, 1.2)	0.734
Cr, *μ*mol/L	57.3 (51.8, 66.3)	53.5 (43.3, 61.3)	0.137
ANA positive, N (%)	13/14	12/14	>0.999
AMA positive, N (%)	9/14	9/14	>0.999
LKM positive, N (%)	0	0	-
LC-1 positive, N (%)	0	0	-
SLA positive, N (%)	0	0	-
Concurrent autoimmune diseases, N (%)	4/14	6 /14	0.695
IgG (g/L)	22.8 (20.2, 25.7)	28.6 (19.7, 32.4)	0.125
IgM (g/L)	2.2 (1.3, 5.7)	2.7 (2.4, 3.0)	0.306
APRI score	3.9 (2.2, 5.3)	6.4 (3.9, 7.2)	0.051
FIB-4 index	6.6 (4.9, 8.7)	11.7 (6.7, 18.9)	0.075
Complications			
Ascites	12/14	10/14	0.648
Variceal bleeding	2/14	4/14	0.648
Prognostic scores			
Child-Pugh score	6.5 (6.0, 7.0)	8.0 (6.0, 8.0)	0.265

*Note*. UDCA, ursodeoxycholic acid; IS, immunosuppressants; TBIL, total bilirubin; ALT, alanine aminotransferase; AST, aspartate aminotransferase; ALP, alkaline phosphatase; GGT, gamma-glutamyl transpeptidase; ALB, albumin; GLB, globulin; ANA, antinuclear antibody; AMA, anti-mitochondrial antibody; LKM, liver–kidney microsomal antibody; LC-1, antibody against liver cytosol type 1 antigen; SLA, soluble liver antigen/liver pancreas antibody; APRI, aspartate aminotransferase to platelet ratio index; FIB-4, fibrosis 4 index.

**Table 2:** Comparison of symptoms between the two groups at baseline.

**Symptoms**	**UDCA-only group (N=14)**	**UDCA+IS group (N=14)**	***P* value**
Jaundice	3 (21.4%)	6(42.9%)	0.420
Ventosity	5(35.7%)	3(21.4%)	0.678
Fatigue	4(28.6%)	3(21.4%)	>0.999
Lower limb swelling	3(21.4%)	4(28.6%)	>0.999
Anorexia	2(14.3%)	3(21.4%)	>0.999
Arthralgia	2(14.3%)	4(28.6%)	0.648
Yellow urine	2(14.3%)	3(21.4%)	>0.999
Abdominal pain	3(21.4%)	2(14.3%)	>0.999
Weight loss	2(14.3%)	1(7.2%)	>0.999
Nausea	1(7.2%)	1(7.2%)	-
None	1(7.2%)	1(7.2%)	>0.999

**Table 3:** Histological features of the decompensated PBC-AIH patients.

**Variables**	**UDCA-only group (N=14)**	**UDCA+IS group (N=14)**	***P* value**
Number of portal areas	10	9	0.541
Severe interface hepatitis	0/14	2/14	0.481
Moderate interface hepatitis	14/14	12/14	0.481
Hepatocyte rosette formation	8/14	10/14	0.695
Lymphoplasmacytic infiltrate	13/14	13/14	>0.999
Emperipolesis	0/14	0/14	-
Bile duct damage	13/14	10/14	0.326
Ductopenia	9/14	6/14	0.449
Bile duct proliferation	11/14	9/14	0.678
Cholestasis	7/14	6/14	>0.999
G0/1/2/3/4 (N)	0/0/2/12/0	0/1/2/7/4	0.374
S0/1/2/3/4 (N)	0/3/4/5/2	0/0/3/8/3	0.117

*Note*. UDCA, ursodeoxycholic acid; IS, immunosuppressants.

**Table 4:** Response to treatment in AIH features after 3, 6, and 12 months of therapy.

**Variables**	**Overall (n=28, %)**	**UDCA-only group (n=14), %**	**UDCA+IS group (n=14), %**	***P* value**
3-month remission	4(14.2%)	0(0%)	4(28.6%)	0.098
6-month remission	6(21.4%)	1(7.1%)	5(35.7%)	0.165
12-month remission*∗*	7(33.3%)^#^	1(9.1%)^##^	6(60.0%)	0.024

*∗*Twenty-one patients were treated for more than 12 months in total.

^#^Eleven patients were treated for more than 12 months in the UDCA-only group.

^##^Ten patients were treated for more than 12 months in the UDCA+IS group.

**Table 5:** Liver-related adverse events in the two groups.

**Variables**	**UDCA-only group (N=14)**	**UDCA+IS group (N=14)**	***P* value**
Adverse events	9/14(64.3%)	2/14(14.3%)	0.018
Severe ascites	4(28.6%)	1(7.1%)	0.326
Variceal bleeding	1(7.1%)	0	>0.999
Liver failure	4(28.6%)	1(7.1%)	0.596
Transplantation/liver-related death	4/14(28.6%)	1/14(7.1%)	0.326

*Note*. UDCA, ursodeoxycholic acid; IS, immunosuppressants.

## Data Availability

All data arising from this study are contained within the manuscript.
